# *N*-(3,5-Dimethyl­phen­yl)-4-nitro­benzene­sulfonamide

**DOI:** 10.1107/S1600536812047502

**Published:** 2012-11-24

**Authors:** U. Chaithanya, Sabine Foro, B. Thimme Gowda

**Affiliations:** a Department of Chemistry, Mangalore University, Mangalagangotri 574 199, Mangalore, India; b Institute of Materials Science, Darmstadt University of Technology, Petersenstrasse 23, D-64287 Darmstadt, Germany

## Abstract

There are two independent mol­ecules in the asymmetric unit of the title compound, C_14_H_14_N_2_O_4_S, in which the dihedral angles between the benzene rings are 56.22 (15) and 58.16 (14)°. In the crystal, N—H⋯O_nitro_ hydrogen bonds link the mol­ecules into zigzag chains running along the *a-*axis direction.

## Related literature

For studies on the effects of substituents on the structures and other aspects of *N*-(ar­yl)-amides, see: Gowda & Weiss (1994 ▶); Shahwar *et al.* (2012 ▶), of *N*-aryl­sulfonamides, see: Chaithanya *et al.* (2012 ▶) and of *N*-chloro­aryl­sulfonamides, see: Shetty & Gowda (2004 ▶). For hydrogen-bonding patterns and motifs, see: Adsmond & Grant (2001 ▶).
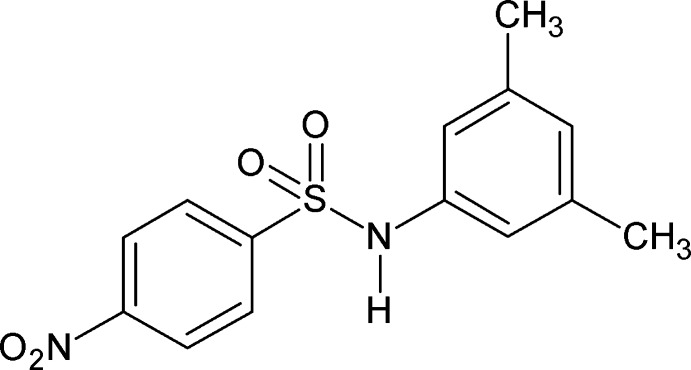


## Experimental

### 

#### Crystal data

C_14_H_14_N_2_O_4_S
*M* *_r_* = 306.33
Orthorhombic, 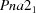

*a* = 14.708 (1) Å
*b* = 7.9410 (7) Å
*c* = 24.741 (2) Å
*V* = 2889.7 (4) Å^3^
*Z* = 8
Mo *K*α radiation
μ = 0.24 mm^−1^
*T* = 293 K
0.38 × 0.30 × 0.24 mm

#### Data collection

Oxford Diffraction Xcalibur diffractometer with a Sapphire CCD detector
Absorption correction: multi-scan (*CrysAlis RED*; Oxford Diffraction, 2009 ▶) *T*_min_ = 0.914, *T*_max_ = 0.944
6693 measured reflections
3714 independent reflections
2624 reflections with *I* > 2σ(*I*)
*R*_int_ = 0.030

#### Refinement

*R*[*F* ^2^ > 2σ(*F* ^2^)] = 0.039
*wR*(*F* ^2^) = 0.074
*S* = 1.00
3714 reflections
390 parameters
3 restraints
H atoms treated by a mixture of independent and constrained refinement
Δρ_max_ = 0.18 e Å^−3^
Δρ_min_ = −0.18 e Å^−3^
Absolute structure: Flack (1983 ▶), 1005 Friedel pairs
Flack parameter: 0.04 (8)

### 

Data collection: *CrysAlis CCD* (Oxford Diffraction, 2009 ▶); cell refinement: *CrysAlis CCD*; data reduction: *CrysAlis RED* (Oxford Diffraction, 2009 ▶); program(s) used to solve structure: *SHELXS97* (Sheldrick, 2008 ▶); program(s) used to refine structure: *SHELXL97* (Sheldrick, 2008 ▶); molecular graphics: *PLATON* (Spek, 2009 ▶); software used to prepare material for publication: *SHELXL97*.

## Supplementary Material

Crystal structure: contains datablock(s) I, global. DOI: 10.1107/S1600536812047502/bt6865sup1.cif

Structure factors: contains datablock(s) I. DOI: 10.1107/S1600536812047502/bt6865Isup2.hkl

Supplementary material file. DOI: 10.1107/S1600536812047502/bt6865Isup3.cml

Additional supplementary materials:  crystallographic information; 3D view; checkCIF report

## Figures and Tables

**Table 1:** Hydrogen-bond geometry (Å, °)

*D*—H⋯*A*	*D*—H	H⋯*A*	*D*⋯*A*	*D*—H⋯*A*
N1—H1*N*⋯O3^i^	0.85 (2)	2.35 (2)	3.129 (5)	152 (3)
N3—H3*N*⋯O8^ii^	0.84 (2)	2.40 (2)	3.168 (5)	152 (3)

Symmetry codes: (i) 
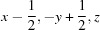
; (ii) 
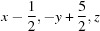
.

**Fig. 1.**
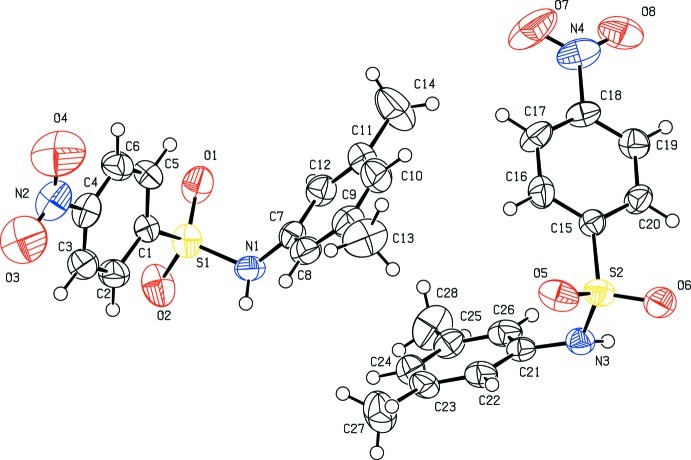
Molecular structure of the title compound, showing the atom labelling scheme and with displacement ellipsoids drawn at the 50% probability level.

**Fig. 2.**
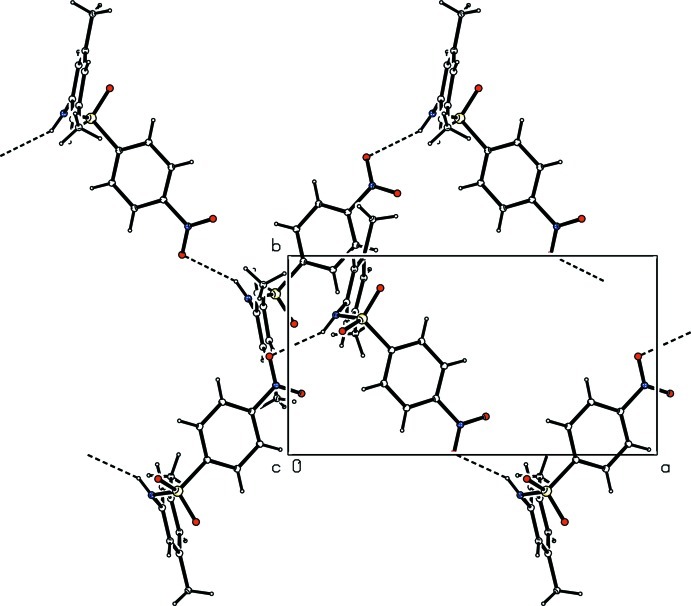
Molecular packing of the title compound with hydrogen bonding shown as dashed lines.

## supplementary crystallographic information

### Comment

As a part of our studies on the substituent effects on the structures and other aspects of *N*-(aryl)-amides (Gowda & Weiss, 1994; Shahwar *et al.*, 2012); *N*-arylsulfonamides (Chaithanya *et al.*, 2012) and *N*-chloroarylsulfonamides (Shetty & Gowda, 2004),in the present work, the crystal structure of *N*-(3,5-dimethylphenyl)-4-nitrobenzenesulfonamide (I) has been determined (Fig. 1).

The asymmetric unit of the structure of (I) contains two crystallographically independent molecules, similar to that observed in *N*-(3,5-dimethylphenyl)-2-nitrobenzenesulfonamide (II) (Chaithanya *et al.*, 2012). The molecules are twisted at the S—N bonds with the torsional angles of -66.67 (38) and -70.56 (39)°, compared to the values of 44.24 (26) and -49.34 (25)° in (II).

The dihedral angles between the sulfonyl and the anilino rings in the two molecules are 56.22 (15)° and 58.16 (14)°, compared to the values of 71.53 (7)° and 72.11 (7)° in (II).

The amide H-atom showed bifurcated intramolecular H-bonding with the O-atom of the *ortho*-nitro group in the sulfonyl benzene ring, generating S(7) motifs and the intermolecular H-bonding with the sulfonyl oxygen atom of the other molecule, generating C(4) motifs (Adsmond *et al.*, 2001).

In the crystal, the intermolecular N–H···O (N) hydrogen bonds (Table 1) link the molecules into zigzag chains running along the a axis. Part of the crystal structure is shown in Fig. 2.

### Experimental

The title compound was prepared by treating 4-nitrobenzenesulfonylchloride with 3,5-dimethylaniline in the stoichiometric ratio and boiling the reaction mixture for 15 minutes. The reaction mixture was then cooled to room temperature and added to ice cold water (100 ml). The resultant solid, *N*-(3,5-dimethylphenyl)-4-nitrobenzenesulfonamide was filtered under suction and washed thoroughly with cold water and dilute HCl to remove the excess sulfonylchloride and aniline, respectively. It was then recrystallized to constant melting point from dilute ethanol. The purity of the compound was checked and characterized by its infrared spectra.

Prism like colourless single crystals of the title compound used in X-ray diffraction studies were grown in ethanolic solution by slow evaporation of the solvent at room temperature.

### Refinement

H atoms bonded to C were positioned with idealized geometry using a riding model with the aromatic C—H = 0.93 Å, methyl C—H = 0.96 Å. The amino H atoms were freely refined with the N—H distance restrained to 0.86 (2) Å. All H atoms were refined with isotropic displacement parameters set at 1.2 *U*_eq_(C-aromatic, N) or 1.5 *U*_eq_(C-methyl).

### Crystal data

**Table:**

C_14_H_14_N_2_O_4_S	*F*(000) = 1280
*M**_r_* = 306.33	*D*_x_ = 1.408 Mg m^−^^3^
Orthorhombic, *P**n**a*2_1_	Mo *K*α radiation, λ = 0.71073 Å
Hall symbol: P 2c -2n	Cell parameters from 1548 reflections
*a* = 14.708 (1) Å	θ = 2.6–27.9°
*b* = 7.9410 (7) Å	µ = 0.24 mm^−^^1^
*c* = 24.741 (2) Å	*T* = 293 K
*V* = 2889.7 (4) Å^3^	Prism, colourless
*Z* = 8	0.38 × 0.30 × 0.24 mm

### Data collection

**Table:**

Oxford Diffraction Xcalibur diffractometer with a Sapphire CCD detector	3714 independent reflections
Radiation source: fine-focus sealed tube	2624 reflections with *I* > 2σ(*I*)
Graphite monochromator	*R*_int_ = 0.030
Rotation method data acquisition using ω scans	θ_max_ = 25.4°, θ_min_ = 2.7°
Absorption correction: multi-scan (*CrysAlis RED*; Oxford Diffraction, 2009)	*h* = −17→6
*T*_min_ = 0.914, *T*_max_ = 0.944	*k* = −9→7
6693 measured reflections	*l* = −16→29

### Refinement

**Table:**

Refinement on *F*^2^	Hydrogen site location: inferred from neighbouring sites
Least-squares matrix: full	H atoms treated by a mixture of independent and constrained refinement
*R*[*F*^2^ > 2σ(*F*^2^)] = 0.039	*w* = 1/[σ^2^(*F*_o_^2^) + (0.0295*P*)^2^] where *P* = (*F*_o_^2^ + 2*F*_c_^2^)/3
*wR*(*F*^2^) = 0.074	(Δ/σ)_max_ < 0.001
*S* = 1.00	Δρ_max_ = 0.18 e Å^−^^3^
3714 reflections	Δρ_min_ = −0.18 e Å^−^^3^
390 parameters	Extinction correction: *SHELXL97* (Sheldrick, 2008), Fc^*^=kFc[1+0.001xFc^2^λ^3^/sin(2θ)]^-1/4^
3 restraints	Extinction coefficient: 0.0037 (3)
Primary atom site location: structure-invariant direct methods	Absolute structure: Flack (1983), 1005 Friedel pairs
Secondary atom site location: difference Fourier map	Flack parameter: 0.04 (8)

### Special details

**Table:**

Experimental. CrysAlis RED (Oxford Diffraction, 2009) Empirical absorption correction using spherical harmonics, implemented in SCALE3 ABSPACK scaling algorithm.
Geometry. All e.s.d.'s (except the e.s.d. in the dihedral angle between two l.s. planes) are estimated using the full covariance matrix. The cell e.s.d.'s are taken into account individually in the estimation of e.s.d.'s in distances, angles and torsion angles; correlations between e.s.d.'s in cell parameters are only used when they are defined by crystal symmetry. An approximate (isotropic) treatment of cell e.s.d.'s is used for estimating e.s.d.'s involving l.s. planes.
Refinement. Refinement of *F*^2^ against ALL reflections. The weighted *R*-factor *wR* and goodness of fit *S* are based on *F*^2^, conventional *R*-factors *R* are based on *F*, with *F* set to zero for negative *F*^2^. The threshold expression of *F*^2^ > σ(*F*^2^) is used only for calculating *R*-factors(gt) *etc*. and is not relevant to the choice of reflections for refinement. *R*-factors based on *F*^2^ are statistically about twice as large as those based on *F*, and *R*- factors based on ALL data will be even larger.

### Fractional atomic coordinates and isotropic or equivalent isotropic displacement parameters (Å^2^)

**Table:**

	*x*	*y*	*z*	*U*_iso_*/*U*_eq_	
S1	0.20029 (7)	0.68335 (15)	0.47849 (4)	0.0495 (3)	
O1	0.2499 (2)	0.8380 (4)	0.48316 (13)	0.0631 (9)	
O2	0.1473 (2)	0.6216 (4)	0.52234 (12)	0.0710 (10)	
O3	0.4490 (3)	0.0058 (6)	0.42894 (19)	0.1052 (15)	
O4	0.5359 (3)	0.1925 (6)	0.3950 (2)	0.1156 (17)	
N1	0.1295 (2)	0.7027 (5)	0.42794 (16)	0.0476 (9)	
H1N	0.094 (2)	0.618 (3)	0.4263 (16)	0.057*	
N2	0.4666 (3)	0.1511 (6)	0.41830 (18)	0.0681 (13)	
C1	0.2802 (2)	0.5258 (5)	0.46126 (14)	0.0381 (9)	
C2	0.2595 (3)	0.3583 (5)	0.47151 (17)	0.0517 (11)	
H2	0.2046	0.3295	0.4877	0.062*	
C3	0.3211 (3)	0.2352 (5)	0.4575 (2)	0.0550 (16)	
H3	0.3089	0.1223	0.4643	0.066*	
C4	0.4008 (3)	0.2829 (6)	0.43324 (19)	0.0481 (12)	
C5	0.4228 (3)	0.4459 (6)	0.42402 (17)	0.0541 (12)	
H5	0.4784	0.4739	0.4085	0.065*	
C6	0.3614 (3)	0.5702 (5)	0.43812 (16)	0.0483 (11)	
H6	0.3751	0.6829	0.4320	0.058*	
C7	0.1582 (3)	0.7666 (5)	0.3761 (2)	0.0422 (12)	
C8	0.1593 (3)	0.6614 (6)	0.33197 (19)	0.0492 (11)	
H8	0.1446	0.5483	0.3364	0.059*	
C9	0.1817 (3)	0.7198 (7)	0.2813 (2)	0.0553 (14)	
C10	0.2049 (3)	0.8896 (7)	0.2769 (2)	0.0626 (13)	
H10	0.2208	0.9325	0.2432	0.075*	
C11	0.2050 (3)	0.9959 (6)	0.3212 (2)	0.0601 (13)	
C12	0.1793 (3)	0.9334 (5)	0.37078 (18)	0.0504 (11)	
H12	0.1763	1.0045	0.4006	0.060*	
C13	0.1821 (3)	0.6099 (7)	0.2319 (2)	0.0839 (17)	
H13A	0.1218	0.6037	0.2173	0.126*	
H13B	0.2024	0.4989	0.2415	0.126*	
H13C	0.2225	0.6568	0.2054	0.126*	
C14	0.2293 (4)	1.1823 (6)	0.3147 (3)	0.103 (2)	
H14A	0.2189	1.2162	0.2779	0.155*	
H14B	0.2921	1.1990	0.3237	0.155*	
H14C	0.1920	1.2487	0.3384	0.155*	
S2	−0.03497 (7)	0.80910 (15)	0.05640 (5)	0.0475 (3)	
O5	0.01620 (19)	0.6581 (3)	0.05262 (13)	0.0618 (8)	
O6	−0.0860 (2)	0.8702 (4)	0.01140 (12)	0.0664 (9)	
O7	0.2958 (3)	1.3138 (6)	0.1386 (2)	0.1138 (18)	
O8	0.2121 (3)	1.4949 (5)	0.09994 (16)	0.0838 (11)	
N3	−0.1086 (2)	0.7845 (4)	0.10462 (16)	0.0446 (10)	
H3N	−0.142 (2)	0.871 (3)	0.1028 (15)	0.054*	
N4	0.2285 (3)	1.3523 (6)	0.11394 (18)	0.0665 (12)	
C15	0.0414 (2)	0.9711 (5)	0.07551 (14)	0.0387 (10)	
C16	0.1215 (3)	0.9299 (5)	0.10135 (17)	0.0530 (11)	
H16	0.1338	0.8183	0.1101	0.064*	
C17	0.1833 (3)	1.0543 (6)	0.1142 (2)	0.0571 (13)	
H17	0.2379	1.0285	0.1313	0.069*	
C18	0.1616 (3)	1.2180 (6)	0.1009 (2)	0.0440 (12)	
C19	0.0830 (3)	1.2609 (5)	0.0751 (2)	0.0486 (14)	
H19	0.0707	1.3723	0.0661	0.058*	
C20	0.0221 (2)	1.1340 (5)	0.06260 (17)	0.0478 (11)	
H20	−0.0323	1.1600	0.0453	0.057*	
C21	−0.0857 (3)	0.7124 (5)	0.1559 (2)	0.0389 (11)	
C22	−0.0684 (3)	0.5420 (5)	0.16021 (18)	0.0468 (11)	
H22	−0.0689	0.4750	0.1294	0.056*	
C23	−0.0504 (2)	0.4699 (5)	0.2103 (2)	0.0481 (11)	
C24	−0.0518 (3)	0.5743 (6)	0.2550 (2)	0.0538 (12)	
H24	−0.0418	0.5267	0.2889	0.065*	
C25	−0.0673 (3)	0.7446 (5)	0.2521 (2)	0.0483 (14)	
C26	−0.0837 (3)	0.8133 (6)	0.2017 (2)	0.0505 (11)	
H26	−0.0935	0.9285	0.1984	0.061*	
C27	−0.0307 (4)	0.2842 (5)	0.2156 (2)	0.0811 (17)	
H27A	−0.0110	0.2407	0.1814	0.122*	
H27B	−0.0849	0.2264	0.2268	0.122*	
H27C	0.0162	0.2674	0.2421	0.122*	
C28	−0.0679 (3)	0.8542 (7)	0.30165 (19)	0.0821 (16)	
H28A	−0.0105	0.9109	0.3048	0.123*	
H28B	−0.0778	0.7856	0.3331	0.123*	
H28C	−0.1157	0.9359	0.2987	0.123*	

### Atomic displacement parameters (Å^2^)

**Table:**

	*U*^11^	*U*^22^	*U*^33^	*U*^12^	*U*^13^	*U*^23^
S1	0.0542 (6)	0.0496 (8)	0.0446 (7)	0.0110 (6)	0.0033 (6)	−0.0041 (7)
O1	0.074 (2)	0.0460 (19)	0.069 (2)	0.0071 (18)	−0.0228 (18)	−0.0130 (19)
O2	0.077 (2)	0.086 (2)	0.0502 (19)	0.0283 (19)	0.0294 (17)	0.0126 (18)
O3	0.102 (3)	0.062 (2)	0.151 (4)	0.034 (3)	0.006 (3)	−0.027 (3)
O4	0.077 (3)	0.117 (3)	0.153 (5)	0.029 (3)	0.042 (3)	−0.034 (3)
N1	0.037 (2)	0.046 (2)	0.060 (3)	−0.0075 (18)	0.0004 (18)	0.003 (2)
N2	0.065 (3)	0.067 (3)	0.072 (3)	0.019 (3)	−0.007 (2)	−0.022 (3)
C1	0.042 (2)	0.036 (2)	0.036 (2)	0.003 (2)	0.0014 (18)	0.0015 (19)
C2	0.044 (2)	0.047 (3)	0.064 (3)	0.000 (2)	0.005 (2)	0.005 (2)
C3	0.054 (3)	0.034 (3)	0.078 (5)	0.002 (2)	−0.008 (3)	0.000 (2)
C4	0.047 (3)	0.052 (3)	0.046 (3)	0.018 (3)	0.000 (2)	−0.011 (2)
C5	0.049 (3)	0.059 (3)	0.054 (3)	−0.003 (3)	0.010 (2)	0.007 (3)
C6	0.054 (3)	0.041 (3)	0.050 (3)	−0.004 (2)	0.004 (2)	0.008 (2)
C7	0.033 (2)	0.044 (3)	0.050 (3)	0.006 (2)	−0.001 (2)	0.002 (2)
C8	0.040 (2)	0.048 (3)	0.059 (3)	−0.001 (2)	−0.001 (2)	−0.002 (3)
C9	0.050 (3)	0.065 (3)	0.051 (4)	−0.001 (3)	−0.002 (3)	−0.006 (3)
C10	0.066 (3)	0.072 (4)	0.050 (3)	−0.001 (3)	0.004 (2)	0.012 (3)
C11	0.066 (3)	0.051 (3)	0.063 (3)	0.000 (2)	−0.005 (3)	0.013 (3)
C12	0.054 (3)	0.044 (3)	0.053 (3)	0.000 (2)	−0.009 (2)	−0.008 (2)
C13	0.076 (3)	0.107 (4)	0.069 (4)	−0.007 (3)	0.011 (3)	−0.026 (3)
C14	0.145 (5)	0.058 (3)	0.107 (5)	−0.010 (4)	0.005 (4)	0.026 (4)
S2	0.0512 (6)	0.0443 (7)	0.0471 (7)	−0.0100 (6)	0.0015 (6)	−0.0028 (7)
O5	0.070 (2)	0.0401 (17)	0.075 (2)	−0.0035 (16)	0.0198 (18)	−0.0141 (18)
O6	0.073 (2)	0.078 (2)	0.0489 (19)	−0.0261 (18)	−0.0172 (16)	0.0081 (18)
O7	0.074 (3)	0.095 (3)	0.173 (5)	−0.004 (3)	−0.053 (3)	−0.046 (3)
O8	0.099 (3)	0.058 (2)	0.094 (3)	−0.029 (2)	0.007 (2)	−0.014 (2)
N3	0.039 (2)	0.041 (2)	0.054 (3)	0.0005 (17)	0.0038 (18)	0.003 (2)
N4	0.057 (3)	0.070 (3)	0.073 (3)	−0.013 (3)	0.005 (2)	−0.036 (3)
C15	0.038 (2)	0.036 (2)	0.042 (2)	0.002 (2)	−0.0009 (19)	−0.0019 (19)
C16	0.056 (3)	0.047 (3)	0.056 (3)	0.006 (2)	−0.008 (2)	0.009 (2)
C17	0.042 (3)	0.063 (3)	0.066 (3)	−0.003 (3)	−0.018 (2)	−0.011 (3)
C18	0.036 (2)	0.043 (3)	0.053 (3)	−0.004 (2)	0.005 (2)	−0.019 (2)
C19	0.051 (3)	0.036 (3)	0.059 (4)	0.005 (2)	0.004 (2)	−0.004 (2)
C20	0.039 (2)	0.044 (2)	0.061 (3)	0.005 (2)	−0.009 (2)	−0.002 (2)
C21	0.032 (2)	0.040 (3)	0.045 (3)	−0.002 (2)	0.0048 (19)	−0.003 (3)
C22	0.046 (2)	0.037 (3)	0.057 (3)	−0.006 (2)	0.003 (2)	−0.008 (2)
C23	0.052 (3)	0.031 (2)	0.062 (3)	−0.005 (2)	−0.001 (2)	0.003 (2)
C24	0.049 (3)	0.064 (3)	0.048 (3)	−0.005 (2)	−0.002 (2)	0.011 (3)
C25	0.044 (3)	0.049 (3)	0.051 (4)	0.001 (2)	−0.003 (3)	−0.009 (2)
C26	0.042 (2)	0.039 (3)	0.070 (4)	−0.004 (2)	0.004 (2)	−0.008 (3)
C27	0.108 (4)	0.051 (3)	0.084 (4)	0.006 (3)	−0.017 (3)	0.007 (3)
C28	0.086 (4)	0.101 (4)	0.059 (4)	0.017 (3)	−0.001 (3)	−0.029 (3)

### Geometric parameters (Å, º)

**Table:**

S1—O2	1.423 (3)	S2—O5	1.419 (3)
S1—O1	1.433 (3)	S2—O6	1.428 (3)
S1—N1	1.634 (4)	S2—N3	1.623 (4)
S1—C1	1.769 (4)	S2—C15	1.772 (4)
O3—N2	1.211 (5)	O7—N4	1.203 (5)
O4—N2	1.216 (5)	O8—N4	1.208 (5)
N1—C7	1.442 (6)	N3—C21	1.433 (6)
N1—H1N	0.849 (18)	N3—H3N	0.843 (18)
N2—C4	1.473 (6)	N4—C18	1.486 (6)
C1—C6	1.371 (5)	C15—C20	1.363 (5)
C1—C2	1.388 (5)	C15—C16	1.379 (5)
C2—C3	1.378 (6)	C16—C17	1.380 (6)
C2—H2	0.9300	C16—H16	0.9300
C3—C4	1.369 (7)	C17—C18	1.378 (6)
C3—H3	0.9300	C17—H17	0.9300
C4—C5	1.354 (5)	C18—C19	1.364 (6)
C5—C6	1.382 (5)	C19—C20	1.383 (5)
C5—H5	0.9300	C19—H19	0.9300
C6—H6	0.9300	C20—H20	0.9300
C7—C12	1.366 (5)	C21—C22	1.380 (5)
C7—C8	1.376 (6)	C21—C26	1.387 (6)
C8—C9	1.376 (7)	C22—C23	1.391 (5)
C8—H8	0.9300	C22—H22	0.9300
C9—C10	1.395 (6)	C23—C24	1.382 (6)
C9—C13	1.502 (7)	C23—C27	1.509 (5)
C10—C11	1.383 (6)	C24—C25	1.373 (5)
C10—H10	0.9300	C24—H24	0.9300
C11—C12	1.376 (6)	C25—C26	1.382 (7)
C11—C14	1.532 (6)	C25—C28	1.504 (7)
C12—H12	0.9300	C26—H26	0.9300
C13—H13A	0.9600	C27—H27A	0.9600
C13—H13B	0.9600	C27—H27B	0.9600
C13—H13C	0.9600	C27—H27C	0.9600
C14—H14A	0.9600	C28—H28A	0.9600
C14—H14B	0.9600	C28—H28B	0.9600
C14—H14C	0.9600	C28—H28C	0.9600
			
O2—S1—O1	120.9 (2)	O5—S2—O6	121.0 (2)
O2—S1—N1	105.49 (19)	O5—S2—N3	107.50 (19)
O1—S1—N1	107.79 (19)	O6—S2—N3	105.3 (2)
O2—S1—C1	107.69 (18)	O5—S2—C15	107.14 (17)
O1—S1—C1	106.67 (18)	O6—S2—C15	107.14 (17)
N1—S1—C1	107.78 (19)	N3—S2—C15	108.30 (19)
C7—N1—S1	121.8 (3)	C21—N3—S2	122.8 (3)
C7—N1—H1N	115 (3)	C21—N3—H3N	121 (3)
S1—N1—H1N	110 (3)	S2—N3—H3N	104 (3)
O3—N2—O4	122.6 (5)	O7—N4—O8	123.2 (5)
O3—N2—C4	118.8 (5)	O7—N4—C18	118.2 (5)
O4—N2—C4	118.6 (5)	O8—N4—C18	118.6 (5)
C6—C1—C2	120.9 (4)	C20—C15—C16	120.8 (4)
C6—C1—S1	119.9 (3)	C20—C15—S2	119.6 (3)
C2—C1—S1	119.2 (3)	C16—C15—S2	119.5 (3)
C3—C2—C1	119.3 (4)	C15—C16—C17	119.9 (4)
C3—C2—H2	120.3	C15—C16—H16	120.0
C1—C2—H2	120.3	C17—C16—H16	120.0
C4—C3—C2	118.5 (4)	C18—C17—C16	117.9 (4)
C4—C3—H3	120.8	C18—C17—H17	121.0
C2—C3—H3	120.8	C16—C17—H17	121.0
C5—C4—C3	122.9 (4)	C19—C18—C17	122.9 (4)
C5—C4—N2	118.6 (4)	C19—C18—N4	118.9 (4)
C3—C4—N2	118.4 (5)	C17—C18—N4	118.2 (4)
C4—C5—C6	118.9 (4)	C18—C19—C20	118.2 (4)
C4—C5—H5	120.5	C18—C19—H19	120.9
C6—C5—H5	120.5	C20—C19—H19	120.9
C1—C6—C5	119.4 (4)	C15—C20—C19	120.2 (4)
C1—C6—H6	120.3	C15—C20—H20	119.9
C5—C6—H6	120.3	C19—C20—H20	119.9
C12—C7—C8	120.6 (5)	C22—C21—C26	120.0 (5)
C12—C7—N1	119.6 (5)	C22—C21—N3	120.2 (4)
C8—C7—N1	119.7 (4)	C26—C21—N3	119.8 (4)
C7—C8—C9	121.4 (5)	C21—C22—C23	120.5 (4)
C7—C8—H8	119.3	C21—C22—H22	119.8
C9—C8—H8	119.3	C23—C22—H22	119.8
C8—C9—C10	117.1 (5)	C24—C23—C22	117.6 (4)
C8—C9—C13	123.1 (5)	C24—C23—C27	121.3 (5)
C10—C9—C13	119.8 (5)	C22—C23—C27	121.1 (4)
C11—C10—C9	121.9 (5)	C25—C24—C23	123.4 (5)
C11—C10—H10	119.0	C25—C24—H24	118.3
C9—C10—H10	119.0	C23—C24—H24	118.3
C12—C11—C10	119.1 (4)	C24—C25—C26	117.8 (5)
C12—C11—C14	120.4 (5)	C24—C25—C28	121.8 (5)
C10—C11—C14	120.5 (5)	C26—C25—C28	120.4 (4)
C7—C12—C11	119.9 (5)	C25—C26—C21	120.8 (4)
C7—C12—H12	120.1	C25—C26—H26	119.6
C11—C12—H12	120.1	C21—C26—H26	119.6
C9—C13—H13A	109.5	C23—C27—H27A	109.5
C9—C13—H13B	109.5	C23—C27—H27B	109.5
H13A—C13—H13B	109.5	H27A—C27—H27B	109.5
C9—C13—H13C	109.5	C23—C27—H27C	109.5
H13A—C13—H13C	109.5	H27A—C27—H27C	109.5
H13B—C13—H13C	109.5	H27B—C27—H27C	109.5
C11—C14—H14A	109.5	C25—C28—H28A	109.5
C11—C14—H14B	109.5	C25—C28—H28B	109.5
H14A—C14—H14B	109.5	H28A—C28—H28B	109.5
C11—C14—H14C	109.5	C25—C28—H28C	109.5
H14A—C14—H14C	109.5	H28A—C28—H28C	109.5
H14B—C14—H14C	109.5	H28B—C28—H28C	109.5
			
O2—S1—N1—C7	178.5 (3)	O5—S2—N3—C21	44.9 (4)
O1—S1—N1—C7	48.1 (4)	O6—S2—N3—C21	175.1 (3)
C1—S1—N1—C7	−66.7 (4)	C15—S2—N3—C21	−70.6 (4)
O2—S1—C1—C6	−152.5 (3)	O5—S2—C15—C20	154.9 (3)
O1—S1—C1—C6	−21.4 (4)	O6—S2—C15—C20	23.6 (4)
N1—S1—C1—C6	94.1 (3)	N3—S2—C15—C20	−89.4 (3)
O2—S1—C1—C2	27.4 (4)	O5—S2—C15—C16	−22.5 (4)
O1—S1—C1—C2	158.5 (3)	O6—S2—C15—C16	−153.7 (3)
N1—S1—C1—C2	−85.9 (3)	N3—S2—C15—C16	93.2 (3)
C6—C1—C2—C3	−0.9 (6)	C20—C15—C16—C17	−0.3 (6)
S1—C1—C2—C3	179.2 (3)	S2—C15—C16—C17	177.1 (3)
C1—C2—C3—C4	−0.7 (7)	C15—C16—C17—C18	0.7 (7)
C2—C3—C4—C5	2.2 (7)	C16—C17—C18—C19	−1.1 (7)
C2—C3—C4—N2	180.0 (4)	C16—C17—C18—N4	−179.1 (4)
O3—N2—C4—C5	176.4 (5)	O7—N4—C18—C19	177.1 (5)
O4—N2—C4—C5	−4.7 (7)	O8—N4—C18—C19	−1.9 (6)
O3—N2—C4—C3	−1.5 (7)	O7—N4—C18—C17	−4.9 (7)
O4—N2—C4—C3	177.4 (5)	O8—N4—C18—C17	176.2 (5)
C3—C4—C5—C6	−2.0 (7)	C17—C18—C19—C20	1.1 (7)
N2—C4—C5—C6	−179.8 (4)	N4—C18—C19—C20	179.1 (4)
C2—C1—C6—C5	1.1 (6)	C16—C15—C20—C19	0.3 (6)
S1—C1—C6—C5	−179.0 (3)	S2—C15—C20—C19	−177.1 (3)
C4—C5—C6—C1	0.3 (6)	C18—C19—C20—C15	−0.7 (6)
S1—N1—C7—C12	−71.1 (5)	S2—N3—C21—C22	−72.3 (5)
S1—N1—C7—C8	112.5 (4)	S2—N3—C21—C26	109.8 (4)
C12—C7—C8—C9	0.2 (6)	C26—C21—C22—C23	1.0 (6)
N1—C7—C8—C9	176.6 (4)	N3—C21—C22—C23	−176.9 (3)
C7—C8—C9—C10	1.2 (6)	C21—C22—C23—C24	0.9 (5)
C7—C8—C9—C13	−178.9 (4)	C21—C22—C23—C27	−179.5 (4)
C8—C9—C10—C11	−0.4 (7)	C22—C23—C24—C25	−2.0 (6)
C13—C9—C10—C11	179.6 (4)	C27—C23—C24—C25	178.4 (4)
C9—C10—C11—C12	−1.6 (7)	C23—C24—C25—C26	1.1 (7)
C9—C10—C11—C14	−179.0 (5)	C23—C24—C25—C28	−179.7 (4)
C8—C7—C12—C11	−2.3 (6)	C24—C25—C26—C21	0.9 (6)
N1—C7—C12—C11	−178.7 (3)	C28—C25—C26—C21	−178.3 (4)
C10—C11—C12—C7	3.0 (6)	C22—C21—C26—C25	−1.9 (6)
C14—C11—C12—C7	−179.7 (4)	N3—C21—C26—C25	176.0 (4)

### Hydrogen-bond geometry (Å, º)

**Table:**

*D*—H···*A*	*D*—H	H···*A*	*D*···*A*	*D*—H···*A*
N1—H1*N*···O3^i^	0.85 (2)	2.35 (2)	3.129 (5)	152 (3)
N3—H3*N*···O8^ii^	0.84 (2)	2.40 (2)	3.168 (5)	152 (3)

Symmetry codes: (i) *x*−1/2, −*y*+1/2, *z*; (ii) *x*−1/2, −*y*+5/2, *z*.
